# Methicillin-Resistant *Staphylococcus aureus* USA300 Latin American Variant in Patients Undergoing Hemodialysis and HIV Infected in a Hospital in Bogotá, Colombia

**DOI:** 10.1371/journal.pone.0140748

**Published:** 2015-10-16

**Authors:** Marylin Hidalgo, Lina P. Carvajal, Sandra Rincón, Álvaro A. Faccini-Martínez, Alba A. Tres Palacios, Marcela Mercado, Sandra L. Palomá, Leidy X. Rayo, Jessica A. Acevedo, Jinnethe Reyes, Diana Panesso, Paola García-Padilla, Carlos Alvarez, Cesar A. Arias

**Affiliations:** 1 Department of Microbiology, Pontificia Universidad Javeriana, Bogotá, Colombia; 2 Molecular Genetics and Antimicrobial Resistance Unit, Universidad El Bosque, Bogotá, Colombia; 3 Dirección de Vigilancia y análisis de Riesgo en Salud Pública, Instituto Nacional de Salud, Bogotá, Colombia; 4 Department of Internal Medicine, Division of Infectious Diseases, University of Texas Medical School at Houston, Houston, Texas, United States of America; 5 Hospital Universitario San Ignacio, Pontificia Universidad Javeriana, Bogotá, Colombia; University of Florida, UNITED STATES

## Abstract

We aimed to determine the prevalence of MRSA colonization and examine the molecular characteristics of colonizing isolates in patients receiving hemodialysis and HIV-infected in a Colombian hospital. Patients on hemodialysis and HIV-infected were prospectively followed between July 2011 and June 2012 in Bogota, Colombia. Nasal and axillary swabs were obtained and cultured. Colonizing *S*. *aureus* isolates were identified by standard and molecular techniques. Molecular typing was performed by using pulse-field gel electrophoresis and evaluating the presence of *lukF*-PV/*lukS-PV* by PCR. A total of 29% (n = 82) of HIV-infected and 45.5% (n = 15) of patients on hemodialysis exhibited *S*. *aureus* colonization. MSSA/MRSA colonization was observed in 28% and 3.6% of the HIV patients, respectively and in 42.4% and 13.3% of the hemodialysis patients, respectively. Staphylococcal cassette chromosome *mec* typing showed that four MRSA isolates harbored the type IV cassette, and one type I. In the hemodialysis group, two MRSA isolates were classified as belonging to the USA300-LV genetic lineage. Conversely, in the HIV infected group, no colonizing isolates belonging to the USA300-Latin American Variant (UDA300-LV) lineage were identified. Colonizing isolates recovered from the HIV-infected group belonged to the prevalent hospital-associated clones circulating in Latin America (Chilean [n = 1] and Pediatric [n = 2]). The prevalence of MRSA colonization in the study groups was 3.6% (HIV) and 13.3% (hemodialysis). Surveillance programs should be implemented in this group of patients in order to understand the dynamics of colonization and infection in high-risk patients.

## Introduction

Bacterial infections are one of the most common causes of hospitalization and mortality among patients infected with the human immunodeficiency virus (HIV) and in patients undergoing hemodialysis due to end-stage renal disease. *Staphylococcus aureus* is deemed an important pathogen causing life-threatening infections in these patients [1,2]. Persistent colonization by *S*. *aureus* is considered to be a significant risk factor for subsequent infections [3]. Approximately 20% of healthy adults are thought to be carriers of *S*. *aureus*, another 30% carry them intermittently and 50% are not carriers [3,4]. The rates of *S*. *aureus* colonization are much higher in immunocompromised patients such as those undergoing hemodialysis [1,2]. It is thought that colonizing isolates are a reservoir for recurrent infection in these patients [5]. The prevalence of nasal colonization with *S*. *aureus* in HIV infected people seems to be in the same range as the general population, with some studies showing somewhat higher rates. Cenizal et al. have suggested that HIV may be an independent risk for *S*. *aureus* nasal colonization [6] and prior studies have indicated that the prevalence of MRSA colonization in HIV-infected patients is between 0% and 17% [2,7,8]. In patients with end-stage renal disease (ESRD) receiving hemodialysis, both MSSA and MRSA colonization are common [9–11]. One of the main reasons for colonization is the increased use of catheters in this population. The annual rate of access-related infection has been estimated to be as high as 10% in patients with indwelling catheters, compared to 1% in patients harboring fistulas or grafts [1,9–11]. Indeed, the use of central venous catheters (CVC) has been identified as the most important risk factor for bloodstream infection in hemodialysis patients [12–14].

While MRSA was originally felt to be a pathogen restricted to hospital settings, it has emerged as an important cause of infection among individuals in the community without prior healthcare exposure [15,16]. While skin and soft tissue infections (SSTIs) are the most common manifestations of community-associated MRSA (CA-MRSA), severe and invasive disease such as necrotizing pneumonia, necrotizing fasciitis, and bacteremia can occur [17]. Patients infected with HIV appear more affected by the CA-MRSA epidemic but it is unclear why HIV-infected patients are at increased risk for MRSA. It has been suggested that antibiotic exposure or immune suppression may be contributing factors [18].

Patients with previous or ongoing health care exposure who develop MRSA infection can be further classified as health care-associated hospital onset (HAHO) or health care-associated community onset (HACO) infections. The HAHO infections develop in the hospital, and the HACO infections develop in the community [19]. In the United States, the majority of CA-MRSA isolates belong to a specific genetic lineage designated USA300 (sequence type [ST] 8). These isolates are the most common cause of bacterial skin and soft tissue infections in the US and responsible for the majority of emergency room visits due to these infections [20,21]. First described in 2005 [22,23], a variant of the MRSA USA-300 (designated USA300 Latin American [LV] Variant) lineage emerged in the northern region of South America causing similar clinical presentations to that of the USA-300. However, unlike USA300, isolates belonging to USA300-LV have replaced the previously dominant hospital-associated clones in Colombia (designated Chilean/Cordobes clone, ST5) in hospitals from the region. However, the reservoir of USA300-LV is unknown. In this study, we aimed to investigate the rates of colonization of USA300-LV among patients from a Colombian hospital that historically have had higher rates of MRSA colonization (HIV-infected and hemodialysis patients) to determine if these patients may serve as reservoir for USA300-LV strains.

## Materials and Methods

### Ethical statement

The study was approved by the institutional review board of Pontificia Universidad Javeriana, Bogota, Colombia.

### Patients

From July 2011 to June 2012, patients undergoing hemodialysis and attending an HIV clinic at a Hospital in Bogotá, Colombia were included in the study. Patients undergoing antibiotic treatment, younger than 18 years old, pregnant women and patients with psychiatric diagnosis were excluded from the study. After an exhaustive explanation of the study purpose, patients who signed the informed consent and completed a questionnaire with demographic and medical history information were included.

### Microbiological and molecular methods

Nasal swab specimens were obtained from both anterior nares and axillar region using a culturette sterile swab. An AIMES transport medium was used to preserve the samples. Samples were plated on blood agar and mannitol salt agar and incubated at 35°C for 24–48 h. Colonies were initially identified as *S*. *aureus* using Gram stain, catalase, DNase test and coagulase (using the Staphylotest agglutination test). MRSA isolates were initially identified by using an oxacillin screen plate method. A *mecA* multiplex PCR was performed to confirm the identity of MRSA isolates [24]. Molecular typing was carried out by pulsed-field gel electrophoresis (PFGE) using *Sma*I restriction endonuclease and electrophoretic patterns interpreted as described by Tenover et al. [25]. Control strains representative of the most common CA and HA-MRSA clones circulating in Colombia were included in the PFGE experiments. Staphylococcal chromosomal cassette *mec* (SCC*mec*) typing was performed following the protocols established by Oliveira *et al*., 2002. PCR amplification of the *lukF*-PV/*ukS*-PV genes encoding PVL was performed in all MRSA isolates as described before [24].

### Antimicrobial susceptibility testing

Minimal inhibitory concentrations (MICs) were determined for 10 antibiotics including, oxacillin, erythromycin, gentamicin, ciprofloxacin, tetracycline, clindamycin, trimethoprim-sulfamethoxazole, vancomycin, chloramphenicol, rifampin and linezolid in all MRSA isolates. MICs were determined following the Clinical and Laboratory Standard Institute recommendations using an agar dilution method [26].

### Data analysis

Data analysis included association level with odds ratio (OR) and the respective confidence intervals at 95%. Statistical analysis was performed with the statistics program SPSS 19.

## Results

### *S*. *aureus* colonization

A total of 283 and 33 HIV-infected and hemodialysis patients were included, respectively. The demographic and clinical characteristics of all patients are shown in Table 1 and Table 2. A total of 29% (82 out of 283) of HIV-infected and 45.5% (15 out 33) of hemodialysis were colonized with *S*. *aureus*. The vast majority of isolates (n = 94) from both patient groups were methicillin-susceptible *S*. *aureus* (MSSA). The most common site of colonization was the nares 77.4% (72 of 94 patients), whereas 23% (22/94) of patients were colonized in axillar areas. The MSSA isolates were susceptible to the majority of antibiotics tested except for erythromycin (17 isolates), tetracyclines (n = 5) and gentamicin (n = 1). Genes encoding the Panton-Valentine leukocidin toxin (PVL) were present in six isolates (5 from the HIV group and 1 from hemodialysis patients).

**Table 1:** General characteristics of HIV-infected patients evaluated in this study.

Characteristic		HIV patientsn = 283 (%)
Gender	Male	241 (85,2)
	Female	42 (14,8)
Age	20–40 years old	176 (62,2)
	> 40 years old	107 (37,8)
Frequency of medical control	Weekly	2 (0,7)
	Monthly	208 (73,5)
	Bimonthly	61(21,6)
	Biannual	4 (1,4)
	Others	8 (2,8)
Infection in 6 months	No	251 (88,7)
	Yes	32 (11,3)

**Table 2:** General characteristics of dialysis patients evaluated in this study.

**Characteristic**		**Hemodialysis patients** n **= 33 (%)**
Gender	Male	22 (66,7)
	Female	11 (33,3)
Age	20–40 years old	8 (24,2)
	> 40 years old	25 (75,8)
Time in renal unit	1 to 71 months	24 (72,7)
	More than 71 months	9 (27,3)
Frequency HD	2 days per week	1 (3)
	3 days per week	32 (97)
Comorbidities (# of patients)	No	3 (9,1)
	Yes	30 (90,9)
	Diabetes (12)	
	Hypertension (5)	
	Cancer (2)	
	Vascular disease (2)	
	Chronic kidney disease and end-stage renal disease (9)	
	Venous catheter	3 (9,1)
	AVF	30 (90,9)
Hospitalization in last 6 months	No	19 (57,6)
	Yes	14 (42,4)
Infection in last 6 months	No	25 (75,8)
	Yes	8 (24,2)

*Notes*: *HD*: *Hemodialysis*, *AVF*: *Arteriovenous fistula*.

*Time in renal Unit*: *71 months (ca*. *6 years) is the average time that most patients*

*spend in the dialysis unit before they have complications including death*

### MRSA colonization

The prevalence of MRSA colonization among HIV-infected and hemodialysis patients was low with only 5 isolates confirmed to be MRSA (3 and 2 in HIV-infected and hemodyalisis patients, respectively; prevalence of 3.7% and 13.3%, respectively) (Table 3). The majority of these isolates (n = 4) were recovered from the nares and only one from the axillar region. Molecular characterization of the isolates indicated that the majority of the MRSA colonizing isolates (n = 4) harbored the SCC*mec* type IV cassette (Table 3). PFGE analyses revealed that 3 of the isolates belonged to the hospital-associated (HA) clones known to circulate in Colombia, namely Pediatric (ST5-MRSA-IV) and Chilean/Cordobes (ST5-MRSA-I) clones which typically exhibit resistance to quinolones, aminoglycosides and macrolide/clindamycin (MLS_B_-type) [27] (Table 3). Two of the MRSA isolates exhibited the typical pattern of MRSA USA300-LV, including the genes encoding PVL and the type IV cassette. In contrast with isolates belonging to HA clones, the two USA300-LV isolates were susceptible to all the antibiotics tested (except oxacillin) (Table 3).

**Table 3:** Characteristics of MRSA strains isolated from the two populations evaluated.

	Interpretation of antibiotic susceptibility													
Population group	OXA	ERY	GEN	CIP	TET	CLI	SXT	VAN	CHL	RIF	LIN	PVL (+/-)	SCCmec type	Clonal identification
HIV infection	R	R	R	R	S	R	S	S	S	S	S	(-)	I	Chilean
HIV infection	R	R	R	R	S	R	S	S	S	S	S	(-)	IV	Pediatric
HIV infection	R	R	R	R	S	R	S	S	S	S	S	(-)	IV	Pediatric
Hemodialysis	R	S	S	S	S	S	S	S	S	S	S	(+)	IV	USA300-LV
Hemodialysis	R	S	S	S	S	S	S	S	S	S	S	(+)	IV	USA300-LV

OXA: oxacillin, ERY: erythromycin, GEN: gentamicin, CIP: ciprofloxacin, SXT: trimethoprim-sulfamethoxazole, VAN: vancomycin, CHL: chloramphenicol, RIF: rifampin, LIN: linezolid

## Discussion

Both groups of patients studied here are at risk of colonization by *S*. *aureus* given their immune-compromised status and other conditions inherent to their clinical condition (i.e., presence of catheters and use of antibiotics among others). [8,28,29]. Previous studies have shown that colonization by *S*. *aureus* may lead to a higher probability of developing infections. Indeed, it has been previously shown that in approximately 65% of the cases, genetic similarity between the colonizing and invasive strains can be demonstrated [3,30].

Interestingly, Popovich et al. reported that the incidence of CA-MRSA skin and soft tissue infections is much higher in HIV-positive patients that HIV-negative controls when followed through time. SSTIs among HIV-negative and among HIV-positive individuals increased significantly from period 1 to period 2 in Cook County Health and Hospitals System Chicago, Illinois [7]. Indeed, within the study period (7 years) the overall incidence of CA-MRSA SSTIs was 16-fold higher among HIV-positive individuals than it was among HIV-negative individuals. On the other hand, in hemodialysis patients, bloodstream infections have been considered an important cause of morbidity and mortality in these patients. For example, a 12 year study with patients on hemodialysis indicated that the overall rate of *S*. *aureus* bloodstream infections was 17.9 per 100 patient-years (range 9.7–36.8) with an MRSA rate of 5.6 per 100 patient-years (range 0.9–13.8) [31].

Recently Wang et al. provided results for understanding the molecular epidemiology of MRSA transmission by demonstrating additional distinctive clinical and molecular differences between HAHO- and HACO-MRSA cases that occur in the hospital and community setting. The study showing patients with HACO-MRSA had a higher proportion of renal failure, hemodialysis, residence in an long-term-care facility, invasive devices in the past 12 months, which could create a predisposition to MRSA acquisition in a community, albeit health care-associated environment [19].

Our study confirms findings from previous reports [1,6,11] that higher rates of *S*. *aureus* colonization are present in HIV-infected (29%) and hemodyalisis patients (45.4%) compared with the general population (20–30%). We found similar rates of colonization among Colombian HIV-infected patients compared to other reports in the literature (29–48%) [32–35]. On the other hand, we found considerable higher proportion of *S*. *aureus* colonization if our patient population in relation to previous surveillance studies (12–42%) [9,12,36,37]. Of note, the rates of MRSA colonization in both patients’ groups (3.7% and 13.3% in HIV-infected and hemodialysis, respectively) are within the ranges reported previously (8–13% and 7–74%, respectively) for the same population groups [12,37,38].

In regard to antibiotic susceptibility, MSSA strains isolated in our study were susceptible to most antibiotics (other than β-lactams) with the exception of macrolides, where rates of resistance of 16.4% and 28.5% in HIV-infected and hemodialysis patients, respectively, were found. Similar rates of macrolide resistance (14.8%) in colonizing nasal isolates of *S*. *aureus* were observed in a previous study performed in Cartagena, Colombia which enrolled healthy medical students [39]. These findings suggest that macrolide resistance genes (*erm*) are highly prevalent in Colombian *S*. *aureus*, a phenomenon that is perhaps driven by macrolide use in our country [40].

Several MRSA clones are distributed worldwide with CA-MRSA strains becoming more prevalent in hospital settings [41]. The majority of MRSA clones circulating in Latin America are related to the five major MRSA international clones (NY/Japan, pediatric, Brazilian, Iberian and Hungarian) [27]. Specifically, two major HA-MRSA clones are circulating in South America, the Brazilian ST239 (SCC*mec*III) and Chilean/Chilean Cordobes (MRSA ST5-I) [42]. Nonetheless, in the northern region of South America, an ST8 CA-MRSA genetic variant designated USA300 Latin American variant seemed to have replaced previous prevalent HA clones [42–44]. This strain has particular genetic characteristics that distinguishes it from the North American USA300 counterpart including the absence of the arginine catabolic mobile element (ACME) and the presence of a novel copper and mercury resistance mobile element (COMER) [44]. Our molecular characterization of colonizing isolates are consistent with the presence of prevalent hospital and community associated genetic lineages with the caveat that only one isolate belonging to the MRSA USA300-LV genetic lineage was found in our patient population (a patient undergoing hemodialysis). Our finding is consistent with the notion that CA and HA genetic lineages are now circulating both in the community and hospital settings. All isolates were susceptible to vancomycin and linezolid, antibiotics that are commonly used in hospitals in Colombia.

Although our study only carrier status is detected in both groups of patients, it is important to note that the increase HACO-MRSA invasive infections highlights the importance of prevention efforts targeted at patients in community care health related risk factors, as well as continued focus on infection control practices in hospitals [19].

Our study has some important limitations. *First*, we only performed a single sampling for each patient and colonization status may change with time. Indeed, Lai *et al*. demonstrated that a screening strategy at two different time points for each evaluated patient increases the detection of colonization with *S*. *aureus* because it may detect the intermittent carrier state [45]. *Second*, our sampling approach was limited to specific anatomical areas (nares and pharynx) and sampling of other areas (e.g. genital and inguinal areas) could also increase colonization detection, particularly in patients with HIV infection (recent reports have shown that MRSA often colonize extranasal regions in these patients) [8].

In conclusion, our study demonstrates substantial rates of colonization by *S*. *aureus* (30–54,5%) in two populations known to be at risk of infection by these microorganisms in Bogotá, Colombia. Our study also suggests the possibility that the USA300-LV strain may have a community reservoir in these patients. Surveillance programs would be important to track the epidemiology of MRSA in high-risk population in Colombia.

## Supporting Information

S1 Table Characteristics of HIV patients colonized with *S*. *aureus*. (DOCX)

S2 Table Characteristics of HIV patients colonized with MRSA. (DOCX)

S3 Table Characteristics of patients undergoing hemodialysis colonized with *S*. *aureus*. (DOCX)

S4 Table Characteristics of patients undergoing hemodialysis colonized with MRSA. (DOCX)
