# Supplementary material for: Methicillin-Resistant Staphylococcus aureus USA300 Latin American Variant in Patients Undergoing Hemodialysis and HIV Infected in a Hospital in Bogotá, Colombia
Source: PLoS One. 2015 Oct 16;10(10):e0140748. doi: 10.1371/journal.pone.0140748 (PMC4608721; doi:10.1371/journal.pone.0140748)
Supplement: S1 Table — (DOCX) [file pone.0140748.s001.docx]

**Table S1. Characteristics of HIV patients colonized with *S. aureus***

| **General characteristics** | | **Colonized *S. aureus* (n= 85)** | | **OR** | **IC (95%)** | ***P*** |
| --- | --- | --- | --- | --- | --- | --- |
| Sex | Male | | 74 (87,05) | 1,249 | 0,596-2,618 | 0,556 |
|  | Female | | 11 (12,94) |  |  |  |
| Age | 20-24 years old | | 62 (72,94) | 1,986 | 1,14-3,46 | 0,015 |
|  | > 40 years old | | 23 (27,05) |  |  |  |
| Frequency of medical control | Weekly/Monthly | | 61 (71,76) | 0,836 | 0,472-1,481 | 0,539 |
|  | Others | | 24 (28,23) |  |  |  |
| Comorbidities | No | | 71 (83,52) | 0,767 | 0,378-1,553 | 0,46 |
|  | Yes | | 14 (16,47) |  |  |  |
| Diseases | Infectious | | 3 (3,52) | 1,145 | 0,230-5,711 | 0,868 |
|  | Chronic | | 11 (12,94) |  |  |  |
| Infection in last  6 months | No | | 74 (87,05) | 0,798 | 0,366-1,738 | 0,57 |
|  | Yes | | 11 (12,94) |  |  |  |
