# Supplementary material for: Methicillin-Resistant Staphylococcus aureus USA300 Latin American Variant in Patients Undergoing Hemodialysis and HIV Infected in a Hospital in Bogotá, Colombia
Source: PLoS One. 2015 Oct 16;10(10):e0140748. doi: 10.1371/journal.pone.0140748 (PMC4608721; doi:10.1371/journal.pone.0140748)
Supplement: S2 Table — (DOCX) [file pone.0140748.s002.docx]

**Table S2. Characteristics of HIV patients colonized with MRSA**

| **Characteristics** | | **MRSA (n= 3)** | **OR** | **IC (95%)** | ***P*** |
| --- | --- | --- | --- | --- | --- |
| Sex | Male | 3 (100) | Ind | - | - |
|  | Female | 0 |  |  |  |
| Age | 20-24 years old | 3 (100) | Ind | - | - |
|  | > 40years old | 0 |  |  |  |
| Frequency of medical control | Weekly/Monthly | 2 (66,66) | 0,78 | 0,067-9,02 | 0,842 |
|  | Others | 1 (33,33) |  |  |  |
| Comorbidities | No | 3 (100) | Ind | - | - |
|  | Yes | 0 |  |  |  |
| Diseases | Infectious | 0 | Ind | - | - |
|  | Chronic | 1 (33,33) |  |  |  |
| Infection in last  6 months | No | 2 (66,66) | 0,278 | 0,23-3,350 | 0,284 |
|  | yes | 1 (33,33) |  |  |  |

Note: Ind: indeterminate
