# Supplementary material for: Methicillin-Resistant Staphylococcus aureus USA300 Latin American Variant in Patients Undergoing Hemodialysis and HIV Infected in a Hospital in Bogotá, Colombia
Source: PLoS One. 2015 Oct 16;10(10):e0140748. doi: 10.1371/journal.pone.0140748 (PMC4608721; doi:10.1371/journal.pone.0140748)
Supplement: S3 Table — (DOCX) [file pone.0140748.s003.docx]

**Table S3. Characteristics of patients undergoing hemodialysis colonized with *S. aureus***

| **Characteristics** | | **Colonized *S. aureus* (n= 18)** | **OR** | **IC (95%)** | ***P*** |
| --- | --- | --- | --- | --- | --- |
| Sex | Male | 12 (66,66) | 1 | 0,234-4,278 | 1 |
|  | Female | 6 (33,33) |  |  |  |
| Age | 31-41 years old | 3 (16,66) | 0,4 | 0,078-2,062 | 0,266 |
|  | > 41years old | 15 (83,33) |  |  |  |
| Time in renal unit | 1 to 71 months | 12 (66,66) | 0,455 | 0,023-8,829 | 0,596 |
|  | More than 71 months | 6 (33,33) |  |  |  |
| Frequency HD | 2 days per week | 0 | Ind | - | - |
|  | 3 days per week | 18 (100) |  |  |  |
| Comorbidities | No | 2 (11,11) | 1,58 | 0,143-21,433 | 0,658 |
|  | Yes | 16 (88,88) |  |  |  |
| Disease | Infectious | 2 (11,11) | 1,58 | 0,143-21,433 | 0,658 |
|  | Chronic | 16 (88,88) |  |  |  |
| Vascular device | Venous catheter | 2 (11,11) | 1,58 | 0,143-21,433 | 0,658 |
|  | AVF | 16 (88,88) |  |  |  |
| Hospitalization in last 6 months | No | 10 (55,55) | 0,833 | 0,208-3,345 | 0,797 |
|  | Yes | 8 (44,44) |  |  |  |
| Infection in last 6 months | No | 14 (77,77) | 1,273 | 0,258-6,273 | 0,767 |
|  | Yes | 4 (22,22) |  |  |  |
| Note: HD: Hemodialysis, AVF: Arteriovenous fistula, Ind: indeterminate | | | | | |
