# Supplementary material for: Methicillin-Resistant Staphylococcus aureus USA300 Latin American Variant in Patients Undergoing Hemodialysis and HIV Infected in a Hospital in Bogotá, Colombia
Source: PLoS One. 2015 Oct 16;10(10):e0140748. doi: 10.1371/journal.pone.0140748 (PMC4608721; doi:10.1371/journal.pone.0140748)
Supplement: S4 Table — (DOCX) [file pone.0140748.s004.docx]

| **Table S4. Characteristics of patients undergoing hemodialysis colonized with MRSA** | | | | | | |
| --- | --- | --- | --- | --- | --- | --- |
| **Characteristics** | | **MRSA (n= 2)** | **OR** | **IC(95%)** | ***P*** |  |
| Sex | Male | 0 | Ind | - | - |  |
|  | Female | 2 (100) |  |  |  |  |
| Age | 31-41years old | 2 (100) | Ind | - | - |  |
|  | > 41years old | 0 |  |  |  |  |
| Time in renal unit | 1 to 71 months | 1 (50) | 0,5 | 0,101-2,477 | 0,392 |  |
|  | More than 71 months | 1 (50) |  |  |  |  |
| Frequency HD | 2 days per week | 0 | Ind | - | - |  |
|  | 3 days per week | 2(100) |  |  |  |  |
| Comorbidities | No | 0 | Ind | - | - |  |
|  | Yes | 2 (100) |  |  |  |  |
| Disease | Infectious | 1 (50) | 15 | 0,485-464,202 | 0,063 |  |
|  | Chronic | 1 (50) |  |  |  |  |
| Vascular device | Venous catheter | 0 | Ind | - | - |  |
|  | AVF | 2 (100) |  |  |  |  |
| Hospitalization in last 6 months | No | 1 (50) | 0,778 | 0,041-14,750 | 0,867 |  |
|  | Yes | 1 (50) |  |  |  |  |
| Infection in last 6 months | No | 2 (100) | Ind | - | - |  |
|  | Yes | 0 |  |  |  |  |

Note: HDL: Hemodialysis, AVF: Arteriovenous fistula, Ind: indeterminate
